# New Books

**Published:** 2009-02

**Artificial Intelligence Methods in the Environmental Sciences**

Sue Ellen Haupt, Antonello Pasini, Caren Marzban, eds.

New York:Springer, 2009. 424 pp. ISBN: 978-1-4020-9118-6, $89.95

**Biofuels: Securing the Planet’s Future Energy Needs**

Ayhan Demirbas,

New York:Springer, 2009. 336 pp. ISBN: 978-1-84882-010-4, $139

**Boreal Forest and Climate Change**

Pertti Hari, Liisa Kulmala, eds.

New York:Springer, 2008. 582 pp. ISBN: 978-1-4020-8717-2, $239

**Censoring Science**

Mark Bowen

New York:Plume, 2009. 324 pp. ISBN: 0-452-28962-8, $16

**Chemical Evolution and the Origin of Life**

Horst Rauchfuss

New York:Springer, 2008. 340 pp. ISBN: 978-3-540-78822-5, $139

**Ecopolis: Architecture and Cities for a Changing Climate**

Paul F. Downton

New York:Springer, 2009. 608 pp. ISBN: 978-1-4020-8495-9, $279

**Emerging Contaminants from Industrial and Municipal Waste**

Damià Barceló, Mira Petrovic, eds,

New York:Springer, 2008. 284 pp. ISBN: 978-3-540-79209-3, $319

**Human Toxicology of Chemical Mixtures**

Harold Zeliger

St. Louis, MO:Elsevier, 2008. 400 pp. ISBN: 978-0-8155-1589-0, $249

**Information Resources in Toxicology**

Philip Wexler, P.J. Hakkinen, Asish Mohapatra, Steven G. Gilbert

St. Louis, MO:Elsevier, 2009. 1,296 pp. ISBN: 978-0-12-373593-5, $199.95

**Integrated Science: New Approaches to Education**

Michael C. Shaw, Michael E. Brint, David Marcey, eds.

New York:Springer, 2009. 148 pp. ISBN: 978-0-387-84852-5, $39.95

**Introduction to Proteomics**

Agnieszka Kraj, Jerzy Silberring, eds.

Hoboken, NJ:John Wiley & Sons, Inc., 2008. 352 pp. ISBN: 978-0-470-05535-9, $89.95

**New Models for Ecosystem Dynamics and Restoration**

Richard J. Hobbs, Katharine Suding

Washington, DC:Island Press, 2008. 512 pp. ISBN: 978-1-59726-185-2, $50

**Nonlinear Regression with R**

Christian Ritz, Jens Carl Streibig

New York:Springer, 2009. 148 pp. ISBN: 978-0-387-09615-5, $54.95

**Phthalates and Cumulative Risk Assessment: The Task Ahead**

Committee on the Health Risks of Phthalates, National Research Council

Washington, DC: National Academies Press, 2008. 208 pp. ISBN: 978-0-309-12841-4, $46

**Principles of Environmental Sciences**

Jan J. Boersema, Lucas Reijnders, eds.

New York:Springer, 2009. 542 pp. ISBN: 978-1-4020-9157-5, $159

**Science and Its History: A Reassessment of the Historiography of Science**

Joseph Agassi

New York:Springer, 2008. 500 pp. ISBN: 978-1-4020-5631-4, $159

**Scientific Data Ranking Methods**

Manuela Pavan, Roberto Todeschini

St. Louis, MO:Elsevier, 2008. 224 pp. ISBN: 978-0-444-53020-2, $195

**The Loom of Life: Unravelling Ecosystems**

Menno Schilthuizen

New York:Springer, 2008. XVI, 168 pp. ISBN: 978-3-540-68051-2, $79.95

**Toxicogenomics: A Powerful Tool for Toxicity Assessment**

Saura Sahu

Hoboken, NJ:John Wiley & Sons, Inc., 2008. 422 pp. ISBN: 978-0-470-51823-6, $190

**Uranium, Mining and Hydrogeology**

Broder J. Merkel, Andrea Hasche-Berger, eds.

New York:Springer, 2008. 958 pp. ISBN: 978-3-540-87745-5, $399

